# A rare initial presentation of giant pheochromocytoma: a case report of acute pulmonary embolism complicated by catecholamine-induced cardiomyopathy

**DOI:** 10.3389/fonc.2026.1772112

**Published:** 2026-04-15

**Authors:** Lujing Zhao, Yanqing Zhang, Yu Zhou, Ruguang Luo, Meng Zhang, Chuanfang Li, Ying Gu

**Affiliations:** Department of Cardiology, Affiliated Hospital of Jining Medical University, Jining, China

**Keywords:** cardiomyopathy, catecholamines, giant tumor, pheochromocytoma, pulmonary embolism

## Abstract

Pheochromocytoma is a rare neuroendocrine tumor characterized by excessive catecholamine secretion, typically presenting with hypertension, headache, palpitations, and sweating. Giant pheochromocytomas (commonly defined as tumors with diameter >4-6cm) are even rarer and may lead to severe cardiovascular complications, including catecholamine-induced cardiomyopathy and thromboembolic events. We report the case of a 76-year-old female who presented with acute pulmonary embolism and heart failure as the initial features of a giant pheochromocytoma, with concurrent deep vein thrombosis and catecholamine- induced cardiomyopathy. Laboratory tests revealed markedly elevated catecholamine metabolites, while imaging confirmed a right adrenal mass measuring 11.1×7.9 cm, along with bilateral pulmonary artery emboli and left lower extremity deep vein thrombosis. After multidisciplinary evaluation, the patient was managed with α- and β-blockers, anticoagulation, and subsequent surgical resection of the tumor. Postoperative follow-up showed significant improvement in cardiac function and complete resolution of thrombotic events. This case demonstrates the importance of including pheochromocytoma in the differential diagnosis of patients with unexplained thromboembolism or cardiovascular collapse, particularly when an adrenal mass is identified on imaging. Early diagnosis, multidisciplinary management, and timely intervention are crucial for favorable outcomes in such complex and life-threatening presentations.

## 1 Introduction

Pheochromocytoma is a rare neuroendocrine tumor that originates from the chromaffin cells of the adrenal medulla. The global pooled incidence is 1.9 cases per million person-years, and the prevalence is approximately 20 cases per million population (1). However, significant geographical variation exists, with incidence rates ranging from 0.54 to 6.6 cases per million person-years across different populations (2–4). The tumor is characterized by excessive catecholamine secretion, which often presents as paroxysmal or persistent hypertension, accompanied by typical symptoms such as headache, palpitations, and diaphoresis (5). In addition to hypertensive crisis, excessive catecholamine production can cause life-threatening cardiovascular complications, including severe hypertension, cardiac arrhythmia, cardiomyopathy, myocardial infarction, and pulmonary edema.

Giant pheochromocytomas are defined as adrenal masses measuring >4–6 cm and account for approximately 8.6%–38.6% of all adrenal tumors (6–8). These tumors can cause marked cardiac injury and increase thrombotic risk through excessive catecholamine release. Currently, few case reports describe giant pheochromocytoma presenting with pulmonary embolism and catecholamine-induced cardiomyopathy, and comprehensive guidance on its diagnosis and management remains limited.

We report a case of giant pheochromocytoma that presented with acute pulmonary embolism and heart failure, accompanied by deep vein thrombosis and catecholamine-induced cardiomyopathy. Through analysis of this case and literature review, we explore its pathogenesis and management strategies to enhance clinicians’ recognition and management of this rare and life-threatening condition.

## 2 Case introduction

A 76-year-old woman was admitted on March 8, 2025, for “paroxysmal dizziness for six months, chest tightness for two weeks, and dyspnea for one day.” Six months before admission, she began to experience paroxysmal dizziness without obvious triggers, accompanied by syncope, fatigue, diaphoresis, and nausea; no diagnosis or treatment was pursued at that time. Two weeks prior to admission, she developed exertional chest tightness, and one day before admission, the chest tightness worsened and dyspnea ensued. She had no significant past medical history, no history of tobacco or alcohol use, and no family history of heart disease. She was taking no medications before presentation and denied use of herbal or dietary supplements. On admission, her heart rate was 120 beats/min and blood pressure was 189/105 mmHg. The abdomen was soft, with a soft palpable mass; there was no regional lymphadenopathy, pain, or other discomfort. The remainder of the physical examination, including the lungs and neurologic examination, showed no abnormalities.

**Figure 1:**
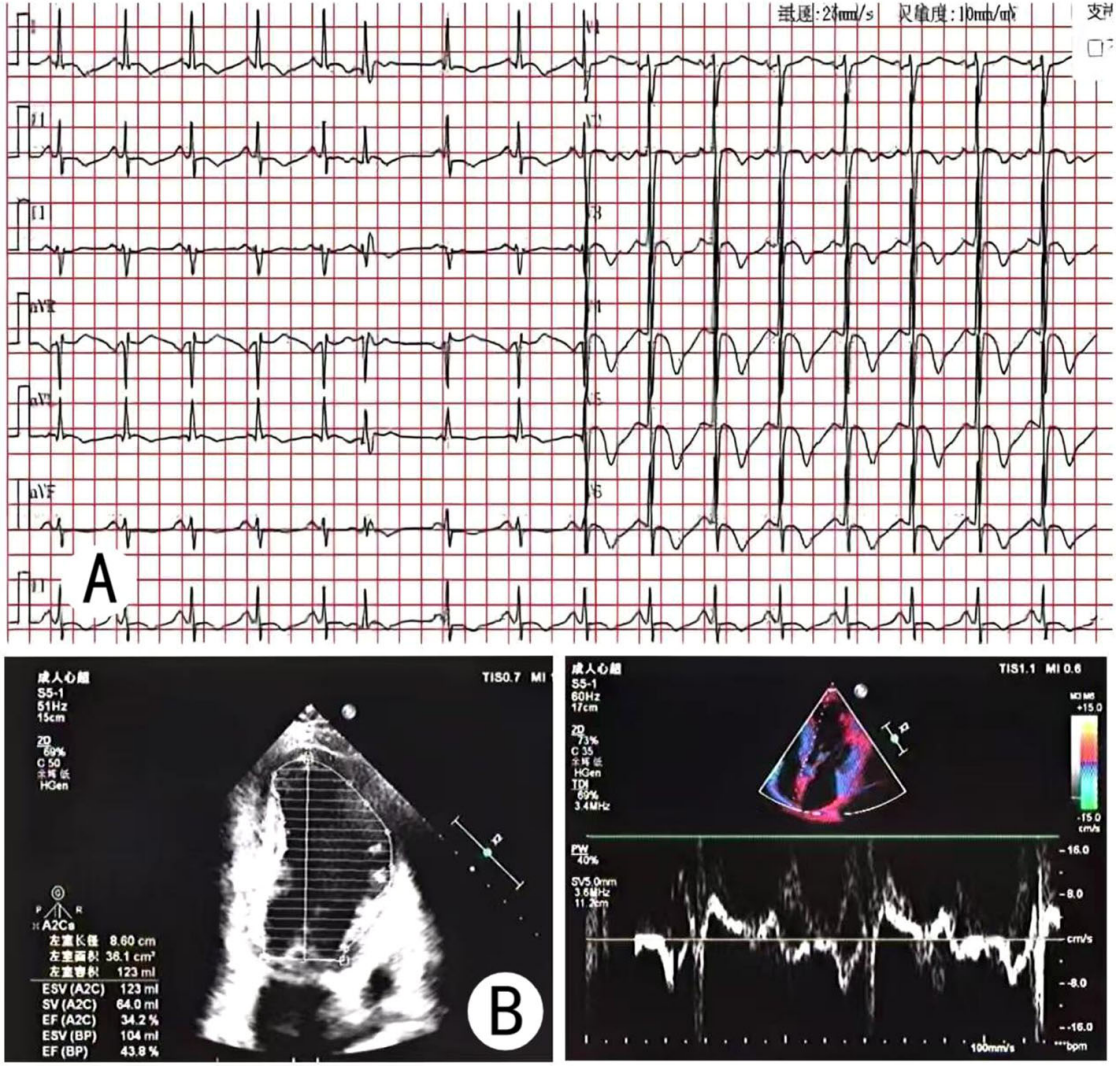
**(A)** 12-lead electrocardiogram shows sinus rhythm with atrial premature beats and T-wave abnormalities. **(B)** The echo-cardiogram indicates: LVEF 43%, segmental wall motion abnormalities (apical segments), and impaired left ventricular diastolic function.

**Figure 2:**
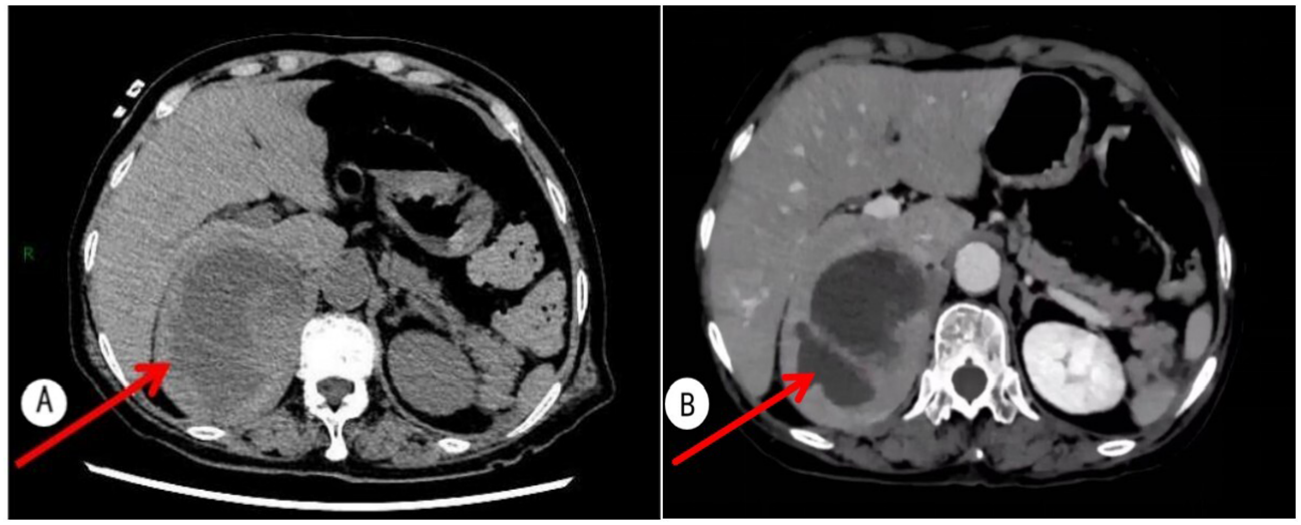
**(A)** Adrenal CT shows a mass lesion with heterogeneous density in the right adrenal region and minimal exudative changes around the right kidney. **(B)** Contrast-enhanced adrenal CT reveals a cystic-solid mass in the right adrenal gland (approximately 11.1 x 7.9 cm in size), highly suspicious for pheochromocytoma, with adrenocortical adenoma/carcinoma also requiring consideration.

**Figure 3:**
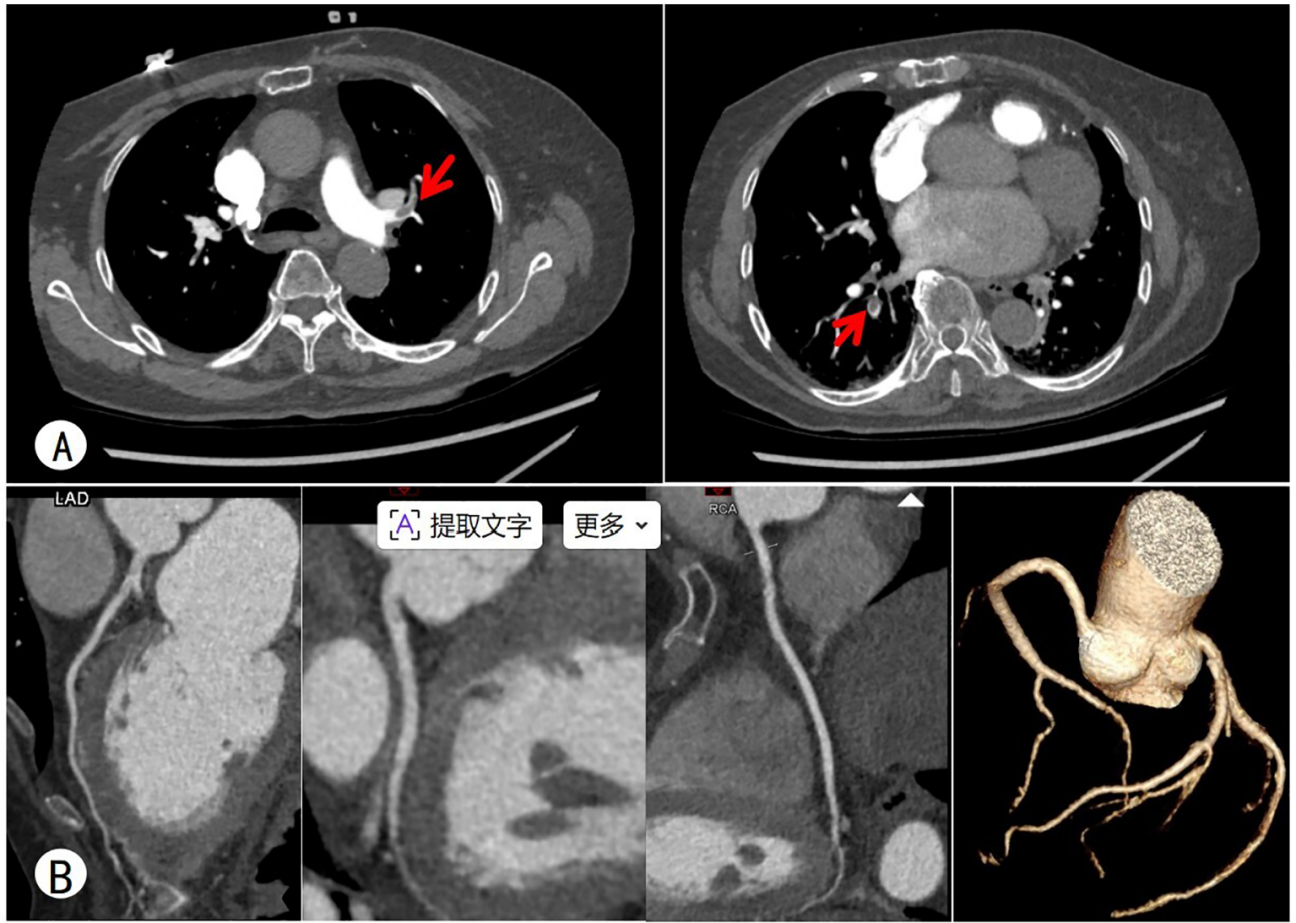
**(A)** Pulmonary artery CTA: Embolism in partial branches of bilateral pulmonary arteries. **(B)** Coronary CTA: approximately 30% stenosis in the LAD and approximately 30% stenosis in the RCA.

Laboratory tests showed marked elevations in markers of myocardial injury and heart failure. Catecholamines and their metabolites greatly exceeded reference ranges. Inflammatory markers on the complete blood count were elevated, and D-dimer was increased. Liver and kidney function tests, thyroid function, electrolytes, antiphospholipid antibodies, protein C, protein S, and antithrombin III activity were normal. At one month postoperatively, the abnormal indicators returned to normal (**Table 1**). Electrocardiogram showed sinus rhythm, atrial premature beats, and T-wave changes (**Figure 1A**), with no typical myocardial infarction patterns. Echocardiography showed a left ventricular ejection fraction (LVEF) of 43% and segmental wall motion abnormality in the apical segment (**Figure 1B**), consistent with decreased cardiac function. Color Doppler ultrasound of the lower limb veins revealed deep vein thrombosis in the left lower limb and local intramuscular venous thrombosis in both calves, which were the primary foci of thromboembolism. Adrenal CT demonstrated a space-occupying lesion in the right adrenal region with uneven density and a small amount of exudation around the right kidney (**Figure 2A**). Adrenal contrast-enhanced CT further verified the lesion was a cystic-solid mass approximately 11.1 × 7.9 cm in size, highly suggestive of pheochromocytoma (**Figure 2B**). Pulmonary CT angiography showed pulmonary embolism involving branch pulmonary arteries bilaterally (**Figure 3A**). Coronary CT angiography showed approximately 30% mild stenosis in the left anterior descending artery and right coronary artery (**Figure 3B**), ruling out severe coronary artery disease as the cause of myocardial injury and chest tightness.

**Table 1:** Laboratory test results.

Test item	2025.03.08 (on admission)	2025.03.10	2025.06.28 (1 month after operation)	Unit	Reference range
Creatine Kinase Isoenzyme MB	7.99 ↑	-	2.10	μg/L	0-5
Myoglobin	96.1 ↑	-	12.1	μg/L	0-70
Troponin I	146.69 ↑	-	24.45	ng/L	0-100
D-Dimer	2.6↑	-	1.2	μg/mL	0-0.5
NT-proBNP	>35000		224	pg/mL	0-300
White Blood Cell Count	15.28 ↑	-	8.6	×10^9^/L	3.5-9.5
Neutrophil Percentage	87.30 ↑	-	67.30	%	40-75
Neutrophil Count	13.33 ↑	-	5.8	×10^9^/L	1.8-6.3
Dopamine	-	5.184↑	0.1	nmol/L	≤0.2
Epinephrine	-	277.576 ↑	0.58	nmol/L	Recumbent position: ≤0.61; Upright position: ≤0.77
Norepinephrine	-	188.181 ↑	3.24	nmol/L	Recumbent position: 0.41-4.43; Upright position: 1.18-10.05
3-Methoxytyramine	-	0.738 ↑	0.12	nmol/L	≤0.13
3-Methoxyepinephrine	-	91.182 ↑	0.331	nmol/L	≤0.5
3-Methoxynorepinephrine	-	55.281↑	0.581	nmol/L	≤0.9

The patient initially presented with paroxysmal dizziness, chest tightness, and dyspnea, and markedly elevated blood pressure was subsequently detected. Laboratory tests showed a pronounced increase in plasma catecholamine metabolites, and imaging identified a large right adrenal mass with radiological features consistent with pheochromocytoma. Therefore, pheochromocytoma was considered the primary diagnosis. The patient also developed dyspnea with elevated D-dimer levels, and pulmonary CT angiography confirmed acute pulmonary embolism; lower-extremity venous ultrasound detected deep vein thrombosis. Echocardiography revealed reduced left ventricular ejection fraction with regional wall motion abnormalities. Given elevated myocardial injury markers and coronary CT angiography showing only mild coronary artery stenosis, acute coronary syndrome was ruled out, and catecholamine-induced cardiomyopathy was considered. Based on the above findings, we concluded that the patient was highly likely to have a large right adrenal pheochromocytoma, catecholamine-induced cardiomyopathy, acute pulmonary embolism, and deep vein thrombosis of the lower extremities.

The patient received the following medication regimen: prazosin hydrochloride 1.5 mg every 8 hours; a valsartan–amlodipine combination once daily; metoprolol succinate extended-release 47.5 mg once daily; furosemide 20 mg once daily; spironolactone 20 mg once daily; and rivaroxaban 15 mg twice daily. After 2 months of systematic treatment, the patient’s heart rate and blood pressure remained stable, and symptoms of heart failure were significantly improved. The surgical and anesthesiology teams developed targeted preoperative prevention and preparation measures which included: 1) continuation of the patient’s baseline medications for alpha (prazosin hydrochloride) and beta (metoprolol succinate) adrenergic blockade, 2) real-time monitoring of blood pressure using an arterial line, and 3) ensured access to emergency rescue medications for catecholamine-induced hypertensive crisis such as phentolamine. A multidisciplinary evaluation was conducted by the endocrinology, cardiology, and vascular surgery departments, among others. After confirmation that the patient’s blood pressure, heart rate, and volume status were adequately controlled, open surgery was performed. The surgery was successfully completed, and the tumor was completely removed. Postoperative pathological examination showed that the tumor cells were arranged in nests and trabeculae. The cell morphology was relatively uniform, mitotic figures were rare, and no significant atypia was observed. The tumor measured 11.1 cm in maximum diameter, meeting the clinical criteria for a giant pheochromocytoma. Immunohistochemistry showed CgA (+), CK (−), GATA-3 (+), Vimentin (partially +), Syn (+), S-100 (+), INSM1 (+), CD56 (+), and Ki-67 (+, 3-8%). Because of the patient’s personal reasons, gene testing related to pheochromocytoma was not performed. Postoperatively, the patient continued metoprolol and rivaroxaban.

At six months postoperatively, follow-up echocardiography showed an LVEF of 55% and an NT-proBNP level of 120 pg/mL. Adrenal CT showed no tumor recurrence. Lower-extremity venous and pulmonary CT angiography showed resolution of the thrombi. The patient reported no dizziness, chest tightness, or dyspnea. Regular follow-up with adrenal imaging and biochemical testing is recommended every six months.

## 3 Discussion

Acute pulmonary embolism is a common and fatal cardiovascular condition. Tumors are an important risk factor for venous thromboembolism (VTE). The incidence of VTE in patients with tumors is 4–7 times that in patients without tumors (9). Approximately 20% of initial VTE events are associated with tumors. However, it is extremely rare for pheochromocytoma to present with acute pulmonary embolism as the first manifestation. The patient in this case had a giant right adrenal pheochromocytoma, catecholamine-induced cardiomyopathy, and deep vein thrombosis of the lower extremities. The clinical manifestations were atypical, which made diagnosis and treatment difficult.

This patient presented with pulmonary embolism as the initial manifestation rather than with typical symptoms, and we attribute this presentation to several factors. First, the tumor measured 11.1×7.9 cm; such a giant pheochromocytoma is rare, and massive, persistent catecholamine secretion provided the core pathological basis for the complications. Second, the tumor was located in the right adrenal region; the local anatomy made the enlarged mass likely to compress the inferior vena cava and iliac vein, obstructing venous return from the lower extremities and promoting venous thrombosis. Third, tumor compression of perirenal tissue induced local exudation and a secondary inflammatory response; this process aggravated local blood stasis and caused endothelial damage, thereby creating favorable conditions for thrombus formation. In addition, excess catecholamines secreted by pheochromocytomas stimulated myocardial β-receptors, inducing calcium overload, oxidative stress, and coronary microcirculatory dysfunction, which led to myocardial stunning, segmental wall-motion abnormalities, and even heart failure (10). Furthermore, it activates the sympathetic nervous system, triggers vasoconstriction and hemodynamic disturbances and directly activates platelets while upregulating levels of coagulation factor VIII and von Willebrand factor, leading to a hypercoagulable state (11, 12). In this case, the marked elevations in white blood cell count and neutrophil percentage indicated tumor-associated inflammation, which may have synergistically enhanced a hypercoagulable state and thus provided an anatomic and pathological basis for thrombosis.

The preferred laboratory tests for diagnosing pheochromocytoma measure plasma free or urinary metanephrine (MN) and normetanephrine (NMN) concentrations. Levels of norepinephrine (NE), epinephrine (E), dopamine (DA), and metabolites such as 3-methoxytyramine (3-MT), homovanillic acid (HVA), and vanillylmandelic acid (VMA) in blood or urine can also be measured concurrently to aid diagnosis (13). The patient’s plasma metanephrine and normetanephrine levels were 91.182 nmol/L and 55.281 nmol/L, respectively; both exceeded the normal range and were consistent with a diagnosis of pheochromocytoma. Adrenal CT is the preferred imaging modality for tumor localization, and its detection rate reaches 90–95% for lesions larger than 1.3 cm. In this case, contrast-enhanced adrenal CT delineated the mass’s size, shape, and radiologic characteristics, providing the basis for localization and diagnosis. For metastatic workup, a whole-body CT was performed and showed no definite distant metastases. Because the lesion was clinically assessed as localized, 68Ga-DOTATATE PET-CT was not performed, and no recurrence was detected at 6-month postoperative follow-up, further supporting the diagnosis of a localized giant pheochromocytoma.

In the differential diagnosis, the patient initially presented with features suggestive of acute coronary syndrome. However, coronary CT angiography showed no significant stenosis or thrombus. Given an elevated D-dimer and biochemical and imaging findings consistent with pheochromocytoma, coronary artery disease was ruled out. Measurement of plasma renin and aldosterone excluded other secondary causes of hypertension, such as primary aldosteronism. Negative antiphospholipid antibodies ruled out antiphospholipid syndrome, and normal activities of protein C, protein S, and antithrombin III excluded hereditary thrombophilia. In clinical practice, pheochromocytoma should be considered in the differential diagnosis of patients with unexplained cardiovascular events or thromboembolism, especially when an adrenal mass is identified.

The case was managed according to established principles for pheochromocytoma treatment. Preoperatively, an alpha-adrenergic blocker was administered to control blood pressure and expand vascular volume, and a beta-adrenergic blocker was added to regulate heart rate and prevent perioperative cardiovascular crises. Concurrently, therapeutic-dose direct oral anticoagulation was promptly initiated for the diagnosed venous thromboembolism. After the patient’s hemodynamics and cardiac function stabilized, the tumor was surgically resected. Postoperatively, cardiac function improved, the thrombosis resolved, and catecholamine levels normalized.

With broader use of imaging and advances in molecular biology, detection rates of Pheochromocytoma and Paraganglioma (PPGLs) have steadily increased, and their genetic features and molecular subtypes have become research priorities. Studies have shown that 30–40% of PPGLs have a hereditary component, making them among the human tumors with the highest genetic susceptibility. Molecular classification divides PPGLs into three major clusters: the pseudohypoxia-related pathway (cluster 1), the kinase-signaling pathway (cluster 2), and the WNT-signaling pathway (cluster 3); tumors in these clusters show marked differences in hormone secretion phenotypes, metastatic risk, and prognosis (14). Although no genetic testing was performed in this case, the patient exhibited marked epinephrine elevation and no radiologic evidence of metastasis; this biochemical phenotype is consistent with the features of cluster 2 tumors as described in the literature. The literature indicates that cluster 2 tumors typically display an adrenergic phenotype and have relatively low clinical aggressiveness. This classification may partially explain the biological basis for the favorable prognosis observed after standard treatment in this patient. Future molecular subtyping of pheochromocytoma will help assess metastatic risk and guide follow-up and may reveal associations between specific subtypes and clinical manifestations (such as a prothrombotic tendency), thereby providing a basis for personalized diagnosis and treatment.

## 4 Conclusion

Pheochromocytoma presenting with thromboembolism as the initial manifestation is rare but can be a fatal complication, especially when tumors are giant and more likely to cause multisystem damage through excessive catecholamine secretion and local compression. In clinical practice, patients with unexplained cardiovascular events or thromboembolism require careful evaluation. If imaging reveals an adrenal mass, prompt biochemical testing for catecholamines is warranted to exclude pheochromocytoma. Successful management of this condition depended on multidisciplinary collaboration, standardized preoperative pharmacological preparation, timely complication management, and complete surgical resection.

## Data Availability

The original contributions presented in the study are included in the article/supplementary material. Further inquiries can be directed to the corresponding author.
